# Sensory Characterization of Cookies With Sugar Replacement by Sweeteners Using the Check‐All‐That‐Apply (CATA) Method

**DOI:** 10.1111/1750-3841.70888

**Published:** 2026-01-28

**Authors:** Igor Henrique Oliveira de Lima, Nathália Letícia Hernandez Brito, Flávia Aparecida Reitz Cardoso, Renata Hernandez Barros Fuchs

**Affiliations:** ^1^ Department of Food Engineering and Chemical Engineering Federal University of Technology – Paraná (UTFPR) Campo Mourão Brazil; ^2^ Postgraduate Program of Food Technology (PPGTA) Federal University of Technology – Paraná (UTFPR) Campo Mourão Brazil; ^3^ Postgraduate Program of Technological Innovations (PPGIT) Federal University of Technology – Paraná Campo Mourão Brazil

**Keywords:** CATA, cookies, luo han guo, sugar reduction, sweetener blends, thaumatin, xylitol

## Abstract

This study investigated the sensory effects of replacing sucrose with blends of xylitol, monk fruit extract (*Siraitia grosvenorii*, rich in mogrosides), and thaumatin in 16 prototype cookie formulations. A consumer‐based check‐all‐that‐apply (CATA) test (*n* = 62 regular cookie consumers) was conducted, followed by Cochran's *Q* test, principal component analysis (PCA), and heatmap visualization of attribute citation frequencies. Balanced sweetener systems were defined as formulations that combined an adequate bulk contribution (xylitol) with high‐intensity sweeteners (monk fruit and thaumatin) at levels that ensured sweetness equivalence while minimizing off‐flavors and structural defects, whereas unbalanced systems lacked this functional complementarity. Twenty‐one sensory attributes were evaluated to characterize differences in appearance, texture, and flavor. Significant differences (Cochran's *Q*, *p* < 0.05) were observed among formulations. Cookies containing unbalanced sweetener ratios exhibited high frequencies of crumbly, sandy, floury, pale, and unpleasant sensory attributes. In contrast, balanced combinations, particularly formulation F16 (xylitol 6 g, monk fruit 0.04 g, and thaumatin 0.03 g per formulation), were characterized by soft, moist, tasty, buttery, and crispy attributes with minimal defects. PCA explained 57.9% of the total variance and revealed three sensory archetypes: underdeveloped, balanced, and overbrowned cookies. The results demonstrate that sugar replacement in cookies depends not only on the intensity of sweetness but also on the mechanistic balance between bulk, browning potential, and flavor modulation provided by blended sweetener systems.

## 1 Introduction

The reduction of added sugars in processed foods has become a global priority, driven by strong public health recommendations and regulatory pressures. Excessive sucrose consumption is associated with obesity, Type 2 diabetes, metabolic dysfunction, and increased cardiovascular risk, leading the World Health Organization (2023) to recommend limiting free sugars to less than 10% of total energy intake, with further health benefits below 5%. Bakery products, particularly cookies, are among the major contributors to daily sugar intake worldwide, making reformulation strategies essential for reducing consumer exposure without compromising product quality. However, reducing sugar in cookies is technologically challenging because sucrose plays multiple functional roles beyond sweetness, influencing dough rheology, spreadability, Maillard browning, color development, crispness, aeration, and flavor release (Manley 2020; Davidson 2018). As a result, sugar replacement often results in cookies that are paler, harder, drier, or less flavorful than conventional products.

To address these limitations, the food industry has increasingly explored combinations of alternative sweeteners rather than relying on a single substitute. Among these, xylitol, luo han guo (monk fruit) extract, and thaumatin stand out due to their natural origin, low caloric contribution, and favorable sweetness profiles. Xylitol, a five‐carbon polyol, provides approximately the same sweetness as sucrose and imparts a characteristic cooling sensation due to its high endothermic heat of solution (O'Donnell and Kearsley 2012). While desirable in some applications, the cooling effect can modify the flavor perception of baked goods. Luo han guo extract contains mogrosides, intense natural sweeteners 200–300 times sweeter than sucrose, but may generate lingering sweetness, herbal notes, or slight bitterness depending on purity and concentration (Zhang et al. 2021). Thaumatin, a sweet‐tasting protein derived from *Thaumatococcus daniellii*, is 1500–3000 times sweeter than sucrose and has a slow onset and long persistence of sweetness, characteristics that can complement polyols and natural extracts when used in blends (Kim and Drake 2017). Previous research suggests that blending sweeteners with different temporal and sensory properties can attenuate sour flavors, reduce bitterness, and create a more cohesive sweetness profile (Carocho et al. 2017; Tan et al. 2019; Mora et al. 2023).

Although many studies have focused on sugar reduction in beverages, dairy products, and tabletop sweeteners, relatively few have investigated the use of blends of xylitol, monk fruit, and thaumatin specifically in cookies. In this category, sugar replacement challenges are particularly pronounced due to the structural role of sucrose. Understanding how these sweeteners interact to modify flavor, sweetness intensity, color, and textural perception is crucial for developing sugar‐reduced bakery products that align with consumer expectations. Among consumer‐based sensory approaches, check‐all‐that‐apply (CATA) has emerged as an efficient method for capturing perception across multiple formulations, providing direct access to consumer language without the need for trained panels (Iwamura et al. 2021, 2022).

Although the combined use of CATA with multivariate tools such as PCA and heatmap visualization has been widely applied to characterize consumer perception in different food matrices (e.g., Tong et al. 2025; Bang et al. 2025), to the authors’ knowledge no previous study has systematically evaluated the combined sensory effects of xylitol, monk fruit extract (*Siraitia grosvenorii*), and thaumatin in cookies, nor interpreted the resulting sensory archetypes in light of their contributions to structure formation, browning reactions, and flavor modulation. Understanding how these sweeteners interact to influence texture, flavor, aroma, and appearance is essential for developing sugar‐reduced cookies that retain the sensory appeal of traditional sucrose‐based products (Kawakami et al. 2025; Castro et al. 2022).

Therefore, this study aimed to provide a comprehensive sensory characterization of sugar‐reduced cookies formulated with blends of xylitol, monk fruit extract, and thaumatin, focusing on the mechanistic links between sweetener composition, sensory archetypes, and consumer perception. Using a Box–Behnken experimental design and the CATA method applied to a consumer panel, we evaluated how different sweetener combinations influence key sensory attributes related to appearance, texture, flavor, and overall impression. By integrating frequency analysis, Cochran's *Q* test, principal component analysis (PCA), and heatmap‐based multivariate analysis, this work offers novel insights into the sensory mechanisms underlying sweetener interactions in cookies. Overall, this research contributes to the rational development of sugar‐reduced bakery products that meet nutritional targets while preserving consumer‐perceived sensory quality.

## 2 Materials and Methods

### 2.1 Ingredients and Experimental Design

Cookies were formulated using the sweeteners thaumatin (Nutramax P10‐GA300, Brazil), monk fruit extract (*S. grosvenorii*, Nutramax LHGE‐201009, Brazil), and xylitol (Essential, Brazil), all supplied by the Department of Food and Chemical Engineering (DAAEQ), Federal University of Technology – Paraná (UTFPR), Campo Mourão, PR, Brazil. The remaining ingredients were purchased from the local market and included Type 1 wheat flour (Coamo, PR, Brazil), unsalted margarine with 80% lipid content (Qualy, SP, Brazil), chemical leavening agent (Royal, SP, Brazil), refined sugar (Alto Alegre, SP, Brazil), refined salt (Cisne, RS, Brazil), and whole eggs (local market, PR, Brazil).

To define the appropriate levels of sweeteners in each formulation, preliminary laboratory trials were conducted to estimate the lower and upper limits of three independent variables: xylitol (0–12 g), monk fruit extract (0–0.08 g), and thaumatin (0–0.05 g). Based on these limits, a Box–Behnken experimental design with three factors and three coded levels (−1, 0, +1) was employed, resulting in 15 experimental formulations and one control formulation prepared with sucrose.

The coded and actual levels of each sweetener, along with the complete experimental matrix, are presented in Table 1. The control formulation was included to serve as a sensory reference for conventional sugar‐sweetened cookies.

**TABLE 1:** Box–Behnken experimental design for the production of cookies with sugar replacement using xylitol, luo han guo, and thaumatin.

Formulations		Coded levels		
		Xylitol (X)	Luo han guo (L)	Thaumatin (T)
1		−1	−1	0
2		−1	1	0
3		1	−1	0
4		1	1	0
5		−1	0	−1
6		−1	0	1
7		1	0	−1
8		1	0	1
9		0	−1	−1
10		0	−1	1
11		0	1	−1
12		0	1	1
13		0	0	0
14		0	0	0
15		0	0	0
16 (control)		0	0	0

		Original variables		
Independents variables	Symbols	−1	0	1
Xylitol (g)	X	0	6	12
Luo han guo (g)	L	0	0.04	0.08
Thaumatin (g)	T	0	0.03	0.05

All formulations were developed to maintain comparable total solids and fat contents, allowing observed differences in sensory perception to be primarily attributed to the sweetener systems rather than to major compositional changes in the base dough.

### 2.2 Cookie Preparation

Sixteen cookie formulations were produced according to the experimental design, including 15 formulations containing different combinations of xylitol, monk fruit extract, and thaumatin, and one sucrose‐based control. All ingredients were weighed using an analytical balance and then manually mixed in a plastic bowl until a homogeneous dough was obtained. Manual mixing was adopted to minimize mechanical variability and avoid excessive gluten development.

After mixing, the dough was rested for 10 min under refrigeration to improve handling consistency. The dough was then rolled on a granite bench using a stainless‐steel rolling pin to a uniform thickness of 5 mm. Cookies were cut using a stainless‐steel circular mold with a diameter of 30 mm, resulting in thin, small‐diameter cookies suitable for short baking times.

Cookies were baked in a preheated electric oven (FTT 240E, Tedesco, Brazil) at 180°C for 6 min. The relatively short baking time was selected due to the reduced thickness and diameter of the cookies, allowing for full baking while enhancing the discrimination of browning differences among formulations, as previously reported in sensory‐oriented cookie studies (Kawakami et al. 2025; Castro et al. 2022).

### 2.3 Sensory Evaluation: Consumer Panel

Sensory evaluation was conducted with a consumer panel composed of adults who reported regular consumption of cookies. Participants were recruited from the university community through verbal invitations and institutional communication channels. Individuals with allergies or intolerances to any cookie ingredient were excluded.

Sensory evaluation was conducted using a consumer panel composed of adults who reported regular consumption of cookies. Participants were recruited from the university community through verbal invitations and institutional communication channels. Individuals with self‐reported allergies or intolerances to any ingredient in cookies were excluded from participation.

A total of 62 consumers participated in the study and completed the evaluation. The number of participants is consistent with recommendations for CATA studies, which indicate that panels of 50–100 consumers are sufficient to provide reliable sensory discrimination and robust frequency‐based statistical analysis, while maintaining feasibility in experimental designs involving multiple samples (Iwamura et al. 2021). The chosen sample size ensured adequate statistical power to detect differences among formulations while minimizing consumer fatigue. This sample size is widely accepted in consumer‐based CATA studies and has been shown to provide stable sensory maps and reliable attribute frequencies, particularly when combined with multivariate analyses such as PCA and heatmap visualization.

Sensory sessions were conducted in a controlled environment under daylight‐type lighting and ambient temperature conditions. Each participant signed an informed consent form prior to the evaluation. The study was approved by the Ethics Committee of the Universidade Tecnológica Federal do Paraná (UTFPR), under CAAE opinion number 88116618.2.0000.5547.

Cookies were served in a monadic, sequential order, according to a balanced presentation design, to minimize order and carryover effects. Samples were coded with random three‐digit numbers and presented on white disposable plates. Consumers evaluated each sample using a CATA questionnaire and were provided with water for palate cleansing between samples (Kawakami et al. 2025; Castro et al. 2022; Iwamura et al. 2021).

### 2.4 CATA Questionnaire and Sensory Attributes

Sensory characterization was performed using the CATA method. A list of 21 sensory attributes was established based on a combination of preliminary laboratory trials, product‐specific knowledge, and previous literature on cookies, sugar reduction, and alternative sweeteners (Lee et al. 2024; Iwamura et al. 2021, 2022). This approach ensured that the attributes were relevant, understandable to consumers, and appropriate for describing differences among formulations.

The selected attributes encompassed the main sensory dimensions of cookies, including appearance (e.g., bright color, dark color, and aerated appearance), texture (e.g., crumbly, sandy, and hard), flavor and taste (e.g., buttery flavor, sweet, refreshing, burnt flavor, and wheat flour flavor), and aftertaste‐related perceptions (e.g., bitter, residual bitterness, and sour flavor), capturing both positive characteristics and potential defects associated with sugar replacement.

For each sample, consumers received a CATA ballot listing all attributes in randomized order. The instruction provided was: “*Please taste the cookie and check all the attributes that you consider applicable to this sample*.” No restriction was imposed on the number of attributes that could be selected. Consumers were instructed to cleanse their palate with water between samples to reduce carryover effects.

### 2.5 Data Collection and Coding

CATA responses were coded in a binary format, where 1 indicated that a given attribute was selected for a sample and 0 indicated that it was not selected. For each formulation, the number of citations for each attribute was counted across all consumers, yielding a frequency table (attributes × formulations). These frequencies were used as the basis for subsequent statistical analyses.

### 2.6 Statistical Analysis

All statistical analyses were conducted using Statistica version 12.0 (StatSoft Inc., Tulsa, OK, USA). A significance level of *p* < 0.05 was adopted for all inferential procedures, unless otherwise indicated. CATA data were analyzed in terms of citation frequencies for each attribute–formulation combination. The analytical strategy combined univariate tests (Cochran's *Q*) with multivariate methods (PCA) and graphical exploration through a heatmap constructed from the CATA frequencies, to summarize the main sensory patterns among formulations.

#### 2.6.1 Cochran's *Q* Test

For each attribute, Cochran's *Q* test was applied to the binary CATA data to determine whether there were significant differences in the frequency of citation among formulations. For this purpose, the data matrix was organized with consumers as blocks and formulations as treatments, and the presence (1) or absence (0) of each attribute as the response. Attributes with significant *Q* values (*p* < 0.05) were considered discriminant and were given particular emphasis in the multivariate interpretation.

#### 2.6.2 Principal Component Analysis

The frequency table (attributes × formulations) was submitted to PCA to visualize the main patterns of variability in the sensory data. Before analysis, frequencies were standardized (using *z*‐scores by attribute) to compensate for differences in overall citation rates among attributes. The covariance (or correlation) matrix of standardized frequencies was then decomposed into principal components. The first two principal components were used to construct biplots that displayed both formulations and attributes, allowing for the interpretation of which descriptors were most strongly associated with each sweetener combination and with the sucrose control.

#### 2.6.3 Heatmap Visualization

To provide an integrated graphical overview of the sensory profiles, a heatmap was constructed using the standardized citation frequencies of all CATA attributes across the 16 formulations. Rows corresponded to sensory attributes and columns to cookie formulations. Color intensity represented the relative frequency of citation for each attribute‐formulation cell, with lighter tones indicating lower frequencies and darker tones indicating higher frequencies.

Standardization (using *z*‐scores by attribute) was applied prior to plotting, allowing attributes with significantly different absolute frequencies to be compared on a common scale. The ordering of formulations and attributes in the heatmap was based on the PCA scores and loadings, respectively, in order to align the heatmap structure with the main sensory dimensions identified by PCA. This graphical representation was used to highlight groups of attributes that tended to co‐occur in the same formulations and to visually distinguish formulations with more defective versus more balanced sensory profiles, without the application of hierarchical clustering algorithms.

#### 2.6.4 Descriptive Statistics

For each formulation, descriptive statistics (absolute frequencies and relative percentages of citations) were calculated for all CATA attributes. These values were summarized in a table (Table 2) to facilitate comparison among sweetener systems and to support the interpretation of PCA and heatmap patterns.

**TABLE 2:** Frequency of citation (%) of selected CATA attributes for the 16 cookie formulations.

Attribute	F1	F2	F3	F4	F5	F6	F7	F8	F9	F10	F11	F12	F13	F14	F15	F16
Bright	1.6	11.3	3.2	4.8	1.6	1.6	1.6	3.2	3.2	3.2	3.2	1.6	0.0	3.2	4.8	4.8
Opaque	32.3	25.8	29.0	32.3	32.3	30.6	30.6	25.8	33.9	25.8	30.6	25.8	27.4	22.6	29.0	21.0
Light color	46.8	46.8	11.3	30.6	40.3	45.2	14.5	9.7	29.0	21.0	12.9	27.4	21.0	29.0	29.0	45.2
Dark color	1.6	1.6	29.0	14.5	8.1	3.2	24.2	33.9	12.9	14.5	30.6	12.9	17.7	16.1	11.3	1.6
Crumbly	22.6	16.1	8.1	11.3	25.8	17.7	3.2	1.6	12.9	21.0	11.3	14.5	14.5	12.9	6.5	14.5
Aerated	8.1	6.5	4.8	4.8	9.7	11.3	4.8	6.5	4.8	9.7	6.5	8.1	6.5	9.7	6.5	6.5
Moist	6.5	9.7	14.5	25.8	8.1	16.1	22.6	22.6	19.4	16.1	17.7	19.4	19.4	16.1	30.6	14.5
Typical cookie texture	4.8	6.5	6.5	11.3	4.8	6.5	6.5	4.8	9.7	12.9	11.3	6.5	9.7	6.5	9.7	16.1
Soft	11.3	29.0	25.8	41.9	19.4	22.6	33.9	46.8	29.0	22.6	35.5	56.5	33.9	32.3	43.5	25.8
Sandy	16.1	11.3	12.9	6.5	21.0	14.5	9.7	3.2	14.5	12.9	9.7	17.7	9.7	9.7	4.8	9.7
Hard	17.7	8.1	9.7	3.2	16.1	8.1	9.7	1.6	12.9	4.8	4.8	6.5	3.2	6.5	3.2	6.5
Crispy	14.5	8.1	6.5	1.6	17.7	12.9	6.5	3.2	9.7	19.4	11.3	6.5	17.7	1.6	0.0	33.9
Residual flavor	6.5	17.7	21.0	12.9	9.7	22.6	4.8	11.3	3.2	11.3	12.9	16.1	8.1	14.5	3.2	4.8
Residual bitterness	9.7	4.8	8.1	4.8	9.7	4.8	6.5	4.8	6.5	3.2	1.6	4.8	3.2	3.2	3.2	1.6
Tasty	9.7	17.7	17.7	24.2	14.5	8.1	25.8	29.0	11.3	21.0	25.8	21.0	30.6	19.4	27.4	33.9
Bad flavor	19.4	6.5	3.2	1.6	8.1	6.5	0.0	1.6	9.7	4.8	3.2	4.8	1.6	3.2	1.6	0.0
Buttery flavor	4.8	9.7	16.1	27.4	14.5	9.7	17.7	11.3	22.6	16.1	22.6	14.5	17.7	17.7	22.6	22.6
Wheat flour flavor	30.6	19.4	8.1	11.3	21.0	21.0	11.3	6.5	25.8	14.5	16.1	14.5	9.7	11.3	17.7	6.5
Sweet flavor	4.8	24.2	35.5	29.0	25.8	27.4	27.4	33.9	6.5	19.4	27.4	25.8	27.4	22.6	27.4	33.9
Burnt flavor	0.0	0.0	6.5	0.0	0.0	0.0	4.8	4.8	1.6	1.6	0.0	0.0	0.0	0.0	0.0	0.0
Refreshing	0.0	0.0	3.2	4.8	0.0	4.8	3.2	1.6	1.6	3.2	3.2	4.8	8.1	1.6	4.8	4.8

This combination of univariate (Cochran's *Q*) and multivariate (PCA and heatmap) approaches provided a comprehensive view of how blends of xylitol, monk fruit, and thaumatin influence the sensory characteristics of sugar‐reduced cookies and their similarity to a conventional sucrose‐sweetened control.

## 3 Results and Discussions

### 3.1 CATA Frequencies and Discriminant Attributes

The raw CATA counts and corresponding percentages are summarized in Table 2. Most attributes showed significant differences among formulations according to Cochran's *Q* test (*p* < 0.05; data not shown), indicating that consumers perceived distinct sensory profiles across the 16 cookies. Attributes such as soft, light color, opaque, sweet flavor, and tasty were frequently selected overall, whereas burnt flavor and refreshing appeared less often and were concentrated in specific formulations.

Formulation F1 was characterized by high citation frequencies for wheat flour flavor (≈31%), sour flavor (≈19%), hard and crumbly textures, and only modest frequencies of sweet and tasty attributes. F5 and F6 also presented elevated frequencies of crumbly, sandy, and wheat flour flavors, suggesting an underdeveloped structure and poor masking of flour notes. In contrast, F16 showed the highest frequencies for tasty (≈34%), sweet flavor (≈34%), typical cookie texture, and crispy, with virtually no sour flavor, burnt flavor, or residual bitterness.

These differences can be mechanistically explained by the functional roles of the sweeteners in the cookie matrix. Formulations dominated by insufficient bulk contribution or unbalanced sweetener ratios were unable to promote adequate starch gelatinization and fat–starch interactions during baking, resulting in crumbly, sandy textures and enhanced perception of wheat flour flavor. In contrast, balanced systems combining xylitol as a bulk provider with small amounts of high‐intensity sweeteners improved water binding, sweetness distribution, and flavor masking, which contributed to softer textures and reduced perception of structural and flavor defects.

These findings mirror prior observations in sugar‐reduced and sugar‐free bakery products: when sucrose is replaced without adequately managing bulk, water distribution, and flavor interactions, cookies tend to become harder, more floury, and less pleasant, whereas optimized formulations using polyols and/or high‐intensity sweeteners can maintain desirable texture and flavor (Winkelhausen et al. 2007; Mushtaq et al. 2010; Ho and Latif 2020; Hajas et al. 2022; Rutkowska and Baranowski 2024). Conversely, formulations combining appropriate levels of bulk sweeteners and high‐intensity sweeteners can achieve sweetness and texture comparable to sucrose controls, as reported for xylitol‐containing cookies and other reduced‐sugar biscuits (Winkelhausen et al. 2007; Mushtaq et al. 2010; Ho and Latif 2020; Hajas et al. 2022; Rutkowska and Baranowski 2024).

### 3.2 Principal Component Analysis

**FIGURE 1:**
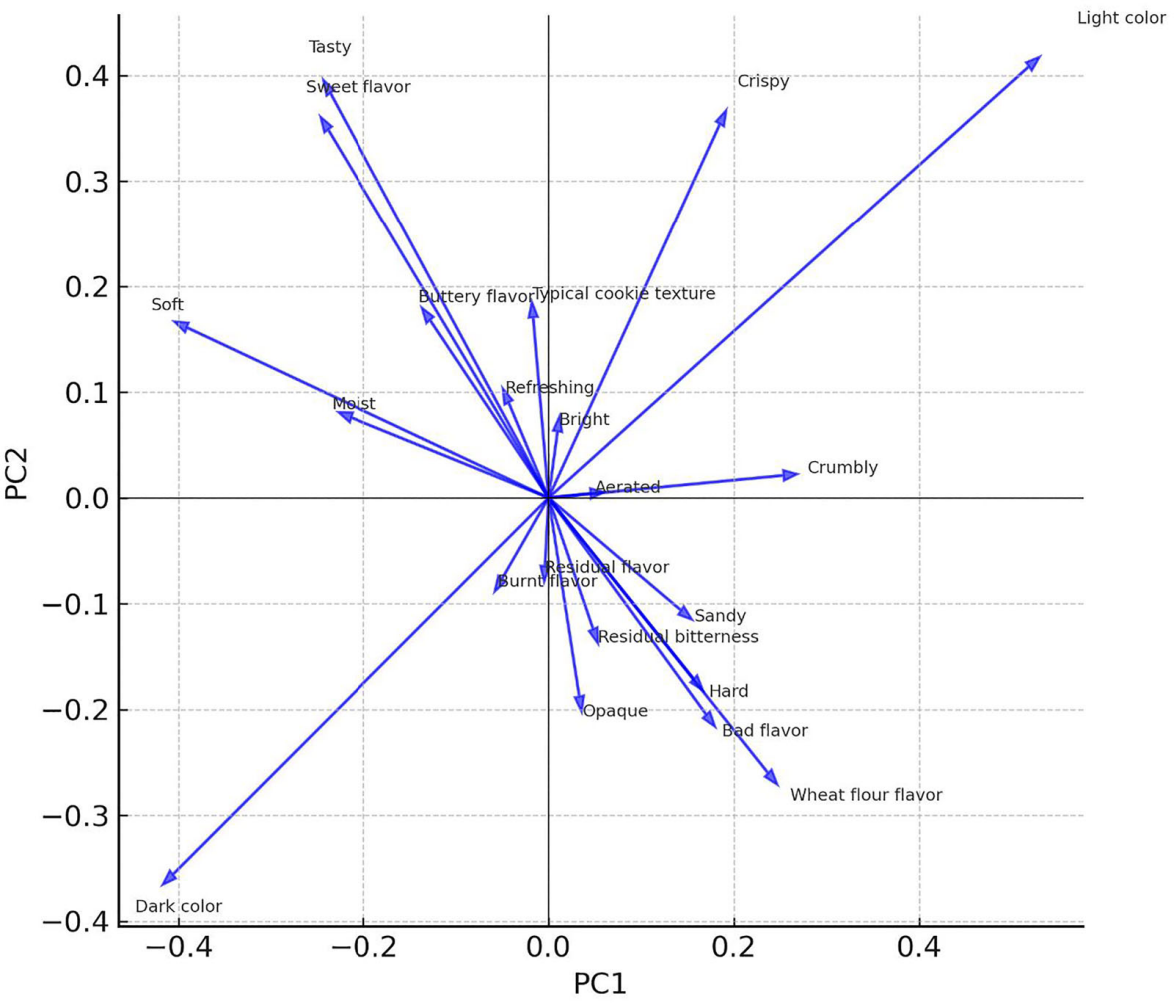
Principal component analysis (PCA) biplot of the 16 cookie formulations based on the frequencies of 21 CATA attributes.

The PCA conducted on the standardized CATA counts revealed that the first two principal components explained 57.9% of the total variance, with PC1 and PC2 accounting for 41.1% and 16.8%, respectively. The joint projection of formulations (scores) and sensory attributes (loadings) is illustrated in the PCA biplot (Figure 1), which enables the simultaneous visualization of sample positioning and their associated descriptors.

Although the first two principal components explained 57.9% of the total variance, which may be considered moderate, this level of explained variance is common in consumer‐based CATA studies due to the binary nature of the data and the inherent heterogeneity of consumer perception. Unlike trained panel profiling, CATA captures individual consumer judgments, which increases dispersion and reduces the proportion of variance captured by the first components. Therefore, PCA was used here as an exploratory tool rather than a predictive model, and its interpretation was complemented by frequency analysis and heatmap visualization to strengthen the robustness of the sensory interpretation.

#### 3.2.1 PC1: From Defective to Well‐Balanced Cookies

PC1 clearly separated formulations associated with defective attributes from those perceived as well‐balanced cookies. In the PCA biplot (Figure 1), hard, crumbly, sandy, wheat flour flavor, light color, opaque appearance, sour flavor, and residual bitterness are located on the positive side of PC1. In contrast, soft, moist, tasty, sweet flavors, buttery flavors, and dark colors lie on the negative side.

Still in Figure 1, formulations F1, F5, F6, and F9 appear with high positive PC1 scores, confirming their characterization as underdeveloped cookies with complex, sandy, and floury textures and evident sour flavors. On the opposite side, F3, F4, F7, F8, F11, F13, F15, and F16 exhibit negative PC1 scores and are associated with soft, moist, tasty, and sweet attributes, indicating more desirable sensory profiles.

This pattern is consistent with previous reports that sugar replacement with polyols and high‐intensity sweeteners can either deteriorate or maintain sensory quality depending on the level and type of sweetener used (Winkelhausen et al. 2007; Mushtaq et al. 2010; Ho and Latif 2020; Hajas et al. 2022; Rutkowska and Baranowski 2024). In particular, xylitol‐based cookies have been shown to retain similar aroma profiles and pleasant textures when properly formulated, whereas inadequate replacement strategies lead to increased hardness and less appealing flavor (Winkelhausen et al. 2007; Mushtaq et al. 2010; Ho and Latif 2020; Hajas et al. 2022; Rutkowska and Baranowski 2024).

#### 3.2.2 PC2: Browning, Burnt Notes, and Crispness

PC2 captured a second dimension related to browning, burnt character, and crispness. In the PCA biplot (Figure 1), dark color, burnt flavor, and residual bitterness tend to be located on the negative side of PC2. In contrast, typical cookie texture and crispy attributes are associated with positive PC2 values. Formulations F3, F7, and F8 are positioned in the lower region of PC2, close to dark color and burnt flavor, confirming that these samples were perceived as more intensely baked and slightly burnt.

This behavior resonates with studies where full or partial sugar replacement by polyols increased browning and the risk of burnt notes due to changes in thermal behavior and moisture distribution during baking (Ho and Latif 2020; Güldane et al. 2022; Rutkowska and Baranowski 2024). In contrast, F10 and F16 appear in the upper region of PC2, associated with typical cookie texture and crispy attributes but not with burnt or bitter notes, implying a more optimal baking level and sweetener balance.

### 3.3 Sensory Clustering and Cookie Archetypes

Based on the patterns observed in the PCA biplot and in the heatmap of CATA citation frequencies, the formulations were grouped into three sensory clusters for interpretation. Although cluster formation is interpretative rather than inferential, this approach is widely adopted in CATA‐based sensory studies to facilitate mechanistic discussion of formulation effects.

**FIGURE 2:**
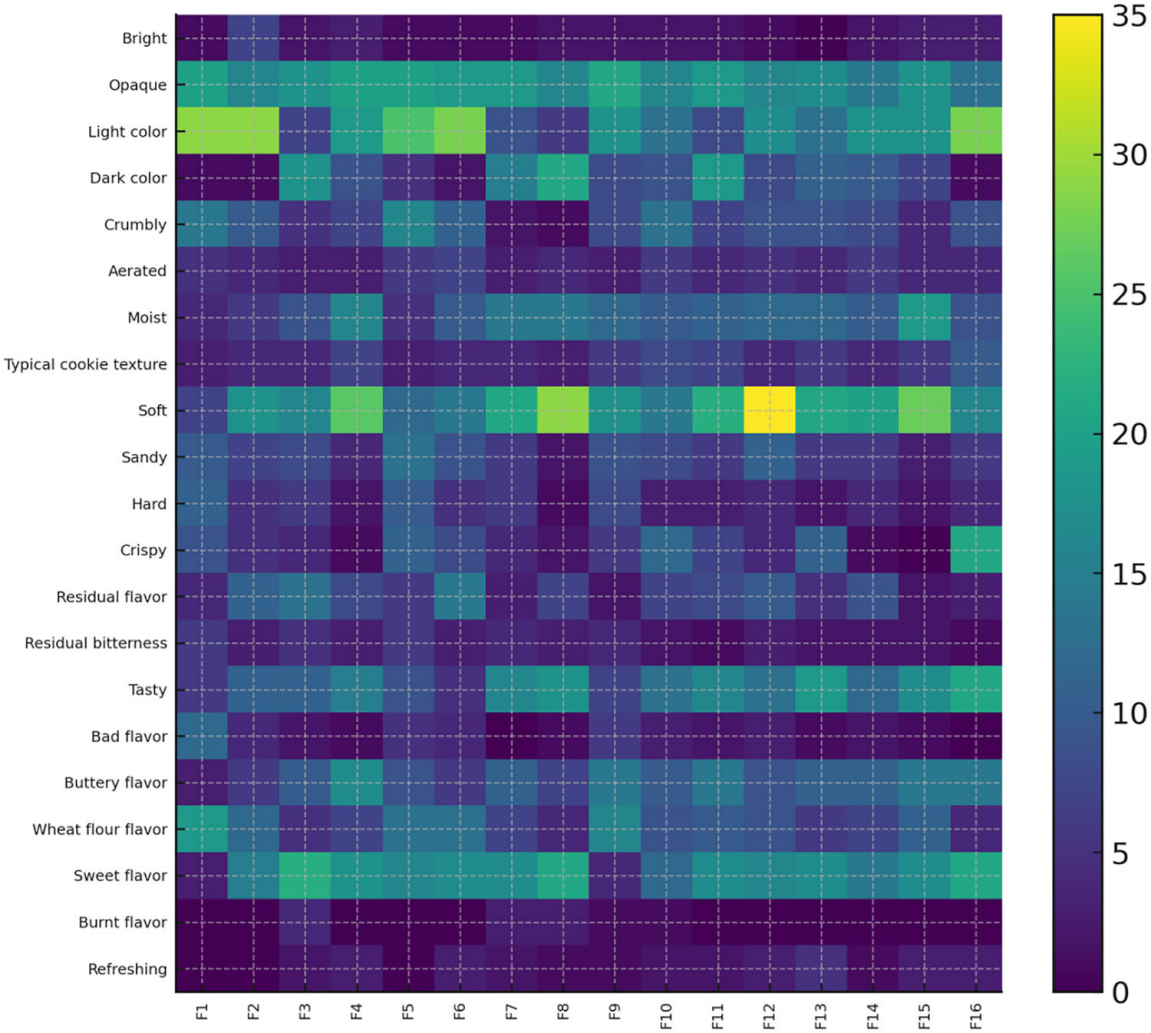
Heatmap of CATA citation frequencies (%) for 21 sensory attributes across the 16 cookie formulations.

The mean citation counts of selected key attributes for each cluster are presented in Table 3, and a heatmap of all attributes across formulations is shown in Figure 2. Cluster 1 (F1, F2, F5, F6, F9) was characterized by high mean counts of light color, wheat flour flavor, crumbly, sandy, and bad flavor, along with relatively low sweet flavor and tasty citations. These formulations exemplify “underdeveloped” cookies, where sweetener combinations fail to promote adequate browning and to mask floury notes.

**TABLE 3:** Mean citation frequency (%) of key CATA attributes within each sensory cluster (*n* = 62 consumers).

Attribute	Cluster 1 (F1, F2, F5, F6, F9)	Cluster 2 (F4, F10–F16)	Cluster 3 (F3, F7, F8)
Light color	36.0	28.5	11.8
Dark color	6.4	13.0	29.0
Crumbly	17.8	11.5	9.1
Sandy	14.9	10.4	12.0
Soft	26.7	35.3	35.5
Moist	14.1	20.5	19.9
Crispy	10.3	11.1	5.3
Tasty	16.0	25.9	24.2
Sweet flavor	17.0	26.4	32.3
Buttery flavor	13.2	19.6	15.0
Wheat flour flavor	22.8	13.0	14.7
Residual bitterness	7.6	3.0	6.5
Bad flavor	13.0	3.0	1.6
Burnt flavor	0.3	0.2	5.2

The “underdeveloped” sensory profile can be attributed to the insufficient participation of the sweetener system in the formation of structure and browning reactions. Reduced sugar availability limits Maillard reactions and caramelization, resulting in a pale color and limited flavor complexity. In addition, inadequate bulk replacement compromises starch gelatinization and fat distribution, which explains the crumbly, sandy texture and the enhanced perception of raw flour notes.

Cluster 2 (F4, F10–F16) comprised the most balanced formulations, showing the highest mean counts of soft, moist, buttery flavor, tasty, sweet flavor, typical cookie texture, and crispy, with low frequencies of defect attributes. Within this cluster, F16 clearly stood out, combining intense positive attributes with almost no sour, burnt, or residual bitterness. This formulation can be considered the best approximation to a “sucrose‐like” cookie among the sugar‐reduced prototypes.

In this cluster, the balance between bulk sweetener (xylitol) and high‐intensity sweeteners appears to optimize both structural and sensory outcomes. Xylitol contributes to water retention and bulk, supporting starch gelatinization and matrix cohesion. Monk fruit extract and thaumatin enhance sweetness intensity and flavor complexity without excessive caloric contribution. This synergy likely explains why formulation F16 most closely resembled sucrose‐based cookies in terms of texture, flavor, and overall sensory quality.

Cluster 3 (F3, F7, F8) exhibited the highest mean counts of dark color, burnt flavor, and residual bitterness, with still high frequencies of sweet and tasty notes. These samples correspond to “overbrowned” cookies in which extensive Maillard reactions and thermal degradation likely contributed to intense color and burnt notes, as previously observed in sugar‐free or polyol‐rich biscuits (Winkelhausen et al. 2007; Mushtaq et al. 2010; Ho and Latif 2020; Hajas et al. 2022; Rutkowska and Baranowski 2024).

The “overbrowned” archetype is consistent with intensified thermal reactions during baking, possibly driven by altered heat transfer and moisture dynamics associated with polyol‐rich formulations. Xylitol and other polyols may accelerate localized browning under certain conditions, increasing the risk of excessive Maillard reactions and thermal degradation, which manifest as dark color and burnt flavor.

These sensory archetypes are in line with CATA studies on cookies, crackers, and diet cakes, which often identify distinct sensory archetypes (e.g., dry/floury, moist/pleasant, and dark/burnt) that reflect formulation and processing differences (Winkelhausen et al. 2007; Mushtaq et al. 2010; Ho and Latif 2020; Hajas et al. 2022; Rutkowska and Baranowski 2024). The present results reinforce that sugar‐reduction strategies based on sweetener blends must carefully control both the level and ratio of components to avoid underdeveloped or overbrowned profiles.

### 3.4 Interpretation in Light of Sweetener Properties

The sensory patterns observed here can be interpreted in light of the known properties of xylitol, monk fruit, and thaumatin. Polyols like xylitol provide moderate sweetness, contribute to bulk, and can participate in browning, but their incomplete fermentability and different heat transfer properties compared with sucrose can lead to modified texture and color (Winkelhausen et al. 2007; Mushtaq et al. 2010; Ho and Latif 2020; Hajas et al. 2022; Rutkowska and Baranowski 2024). Monk fruit sweetener, rich in mogrosides, offers intense sweetness with minimal calories and has been successfully applied in beverages and bakery products, although its sensory impact depends on blending with bulk sweeteners to avoid overly intense or unbalanced sweetness (Zhang et al. 2021; Mora et al. 2023; Carocho et al. 2017). Thaumatin, in turn, is not only extremely sweet but also recognized as a flavor enhancer and bitterness masker, improving overall flavor quality when combined with other sweeteners (FDA 2014; Maluly et al. 2020; Martyn et al. 2018; Carocho et al. 2017). Recent reviews on natural sweeteners and taste modulators corroborate that specific sweetener blends can enhance sweetness while minimizing bitter or metallic off‐tastes, supporting the use of mixtures rather than single sweeteners (An et al. 2025; O'Connor et al. 2021). The current CATA data support this view: formulations that presumably relied too heavily on a single sweetener (or on unbalanced ratios) belonged to the “underdeveloped” or “overbrowned” clusters, whereas formulations combining more appropriate proportions of xylitol, monk fruit and thaumatin, particularly F16, achieved a sensory profile characterized by sweet, tasty, buttery, soft and crispy attributes and very low incidence of defects.

Moreover, the use of consumer‐based CATA rather than trained panel profiling aligns with recent methodological trends that favor rapid sensory tools for product development and reformulation, especially when consumer perception is the target (Ares and Jaeger 2013, 2015; Dooley et al. 2010; Tiepo et al. 2020). The combination of CATA with PCA and heatmap, as demonstrated here, provided a clear and nuanced picture of how sweetener blends reshape the sensory landscape of cookies.

Taken together, these findings suggest that sugar replacement in cookies should not be approached solely as a matter of sweetness equivalence, but rather as a system‐level reformulation challenge. The emergence of underdeveloped, balanced, and overbrowned sensory archetypes highlights that both insufficient and excessive contributions of specific sweeteners can negatively affect structure, flavor, and appearance. A rational formulation strategy should therefore prioritize functional balance, combining bulk provision, controlled browning potential, and flavor modulation to achieve sucrose‐like sensory quality in baked products.

These results suggest that sucrose replacement in baked goods should follow a balance‐based formulation framework, rather than a single‐sweetener substitution approach, in which bulk provision, sweetness modulation, and browning potential are simultaneously optimized.

## 4 Conclusions

The comprehensive sensory evaluation revealed that sugar replacement in cookies is viable when sweeteners are combined in balanced proportions that simultaneously address sweetness intensity, bulk contribution, browning behavior, and flavor modulation. Although several formulations exhibited defects such as pale color, floury taste, excessive hardness, or burnt notes, others—particularly formulation F16—achieved a sensory profile remarkably close to that of a sucrose‐based cookie. These differences demonstrate that successful sugar reduction in baked products depends not only on achieving sweetness equivalence but also on reproducing the structural and thermal roles of sucrose within the cookie matrix.

The integration of consumer‐based CATA with multivariate tools (PCA and heatmap analysis) enabled a robust interpretation of how different sweetener systems reshape sensory perception, leading to the identification of three distinct cookie archetypes: underdeveloped, balanced, and overbrowned. These archetypes reflect underlying formulation‐driven mechanisms, in which insufficient bulk replacement compromises starch gelatinization and flavor development, while excessive or unbalanced sweetener contributions may intensify browning reactions and generate burnt or bitter notes.

Sweetness intensity, flavor masking capacity, browning potential, and textural contribution varied markedly among the sweeteners evaluated, reinforcing the importance of blend optimization rather than reliance on single sweeteners. Thaumatin and monk fruit extract acted as effective sweetness enhancers and bitterness modulators, whereas xylitol played a critical role in providing bulk, supporting structure formation, and moderating browning. Together, these findings support a formulation principle in which the rational combination of bulk sweeteners and high‐intensity sweeteners is essential to achieve sucrose‐like sensory quality in sugar‐reduced cookies.

Overall, this study contributes to the development of a more systematic framework for sugar substitution in bakery products, highlighting that balanced sweetener systems can reproduce desirable sensory attributes while minimizing defects. Future work should include instrumental texture and color measurements, modeling of starch and water interactions, volatile compound profiling, and consumer liking studies with larger and more diverse populations. Such approaches will further validate the proposed sensory archetypes and support the development of predictive formulation strategies applicable to a wide range of baked goods.

## Author Contributions

**Igor Henrique Oliveira de Lima**: conceptualization, methodology, project administration, data curation. **Nathália Letícia Hernandez Brito**: conceptualization, project administration, methodology, data curation. **Flávia Aparecida Reitz Cardoso**: methodology, writing – review and editing, writing – original draft. **Renata Hernandez Barros Fuchs**: conceptualization, methodology, supervision, project administration, writing – review and editing, writing – original draft.

## Conflicts of Interest

The authors declare that they have no conflicts of interest.

## Ethics Statement

The sensory evaluation protocol involving human participants was conducted in accordance with the ethical standards of the institutional research committee and with the Declaration of Helsinki. All participants were adults who voluntarily agreed to participate and provided written informed consent prior to the sensory sessions. The study was reviewed and approved by the Ethics Committee of the Universidade Tecnológica Federal do Paraná (UTFPR), under CAAE number 88116618.2.0000.5547.
